# Association of lncRNA *THRIL, HOTAIR* genes variations and expression levels with pulmonary tuberculosis

**DOI:** 10.1186/s12920-023-01770-x

**Published:** 2023-12-12

**Authors:** Li-Jun Wang, Rui Li, Tian-Ping Zhang, Hong-Miao Li

**Affiliations:** 1 Department of Infectious Diseases, The First Affiliated Hospital of Anhui Medical University, Hefei, Anhui China; 2 Department of Nosocomial Infection Management, The First Affiliated Hospital of Anhui Medical University, Hefei, China; 3 Division of Life Sciences and Medicine, The First Affiliated Hospital of USTC, University of Science and Technology of China, Hefei, Anhui China; 4 Department of Epidemiology and Biostatistics, School of Public Health, Anhui Medical University, Hefei, China

**Keywords:** Pulmonary Tuberculosis, lncRNA, *HOTAIR*, *THRIL*, Genes variation

## Abstract

**Background:**

Long non-coding RNA (lncRNA) has been implicated in the pathogenesis of pulmonary tuberculosis (PTB). This study aims to investigate the involvement of lncRNA *THRIL* and *HOTAIR* gene single nucleotide polymorphisms (SNPs) and their expression levels in PTB susceptibility.

**Methods:**

A total of 456 PTB patients and 464 healthy controls participated in our study. we genotyped six SNPs of *THRIL* and *HOTAIR* genes using an improved multiple ligase detection reaction (iMLDR). Additionally, real-time reverse-transcriptase polymerase chain reaction was employed to detect the expression levels of THRIL and HOTAIR in peripheral blood mononuclear cells (PBMC) from 78 PTB patients and 84 healthy controls.

**Results:**

No significant differences in allele and genotype frequencies were observed for *THRIL* rs1055472, rs11058000, and *HOTAIR* rs12427129, rs1899663, rs4759314, and rs7958904 polymorphisms between PTB patients and healthy controls (all *P* > 0.05). Moreover, genotype frequencies of all SNPs did not show any association with PTB susceptibility in the dominant–recessive model. However, the frequencies of rs7958904 CC genotype and C allele in the *HOTAIR* gene were significantly correlated with leukopenia in PTB patients. Furthermore, the expression levels of the *HOTAIR* gene were significantly elevated in PTB patients compared to controls.

**Conclusions:**

Our study indicates that *THRIL* and *HOTAIR* gene SNPs might not contribute to PTB susceptibility, while the level of *HOTAIR* was increased in PTB patients.

## Introduction

Tuberculosis (TB) caused by the bacillus *Mycobacterium tuberculosis* (MTB), remains a significant global health challenge, leading to substantial morbidity and mortality worldwide [1]. The 2021 global tuberculosis report from the World Health Organization revealed an estimated 9.87 million new TB cases in 2020 with an incidence rate of 127/10,000 and a fatality rate of 15% [2]. Pulmonary tuberculosis (PTB), the predominant form of TB, exerts a severe impact on the well-being of millions and imposes a substantial economic burden on society [3]. Previous studies have highlighted that only a small percentaget (5–10%) of individuals infected with MTB progress top active PTB [4]. This observation underscores the multifactorial nature of PTB development, including environmental conditions, immune status, and host genetic variations [5, 6]. Numerous investigations have associated genetic variations in specific genes, such as vitamin D receptor, human leukocyte antigen, C-type lectin-like domain, and ADP-ribosylation factor with SH3, Ankyrin repeat, and PH domain (HLA, CLEC4E, and ASAP1, respectively), with the risk of PTB development [3, 7–9].

Long non-coding RNA (lncRNA), characterized by more than 200 nucleotides and lacking protein-coding potentials [10], has emerged as a significant player in various biological processes, including epigenetic modification, transcriptional regulation, post-transcriptional processing and translation regulation [11, 12]. Recent studies have implicated genetic variations of lncRNAs, such as CASC8, HNF1B3:1, AC0007128.1 and others, in the occurrence and progression of PTB [13–15]. Our previous investigation demonstrated a correlation between lncRNA NEAT1 gene polymorphisms and PTB development [16]. However, studies exploring the relationship between lncRNA genes variation and PTB susceptibility remain limited.

HOX Transcript Antisense RNA (HOTAIR), the pioneering lncRNA that resides on chromosome 12, spanning 2.2 kb, is established as a regulator of epigenetic mechanisms [12]. Conversely, LncRNA THRIL (Tumor Necrosis Factor Alpha- and Heterogenous Nuclear Ribonucleoprotein- [TNFα- and hnRNPL-, respectively] related immunoregulatory lincRNAs) participates in the innate immune response and inflammatory diseases by modulating TNFα expression through its interaction with hnRNPL [12, 17]. Prior investigations have linked polymorphisms in the *HOTAIR* and *THRIL* genes polymorphisms to various diseases, including rheumatoid arthritis, cancers, preeclampsia (PE) and others [18–21]. However, no research has explored the potential association between variations in these genes and susceptibility to PTB. Thus, this study aims to assess the relationship between lncRNA *THRIL* and *HOTAIR* gene polymorphisms and their expression levels in the context of PTB.

## Materials and methods

### Samples

For this research, 456 PTB patients and 464 healthy controls, all of Chinese nationality, were recruited from the Department of Tuberculosis at Anhui Chest Hospital to investigate the link between *THRIL* and *HOTAIR* gene polymorphisms and PTB susceptibility. Additionally, 78 PTB patients and 84 healthy controls were included to examine gene expression levels. The average age of the 84 controls (56 males and 28 females) was 49.39 ± 17.00 years, and for the 78 patients (51 males and 27 females), it was 49.83 ± 18.59 years. All diagnoses were made by specialists based on suspicious clinical symptoms, chest radiography, sputum and/or bronchoalveolar lavage fluid MTB culture, microscopy for acid-fast bacilli (AFB), and the response to anti-TB treatment. Exclusion criteria encompassed human immunodeficiency virus (HIV), hepatitis, malignancies, and/or immune-compromised conditions. Healthy controls, selected from the same district health examination center, exhibited asymptomatic negative sputum smears and cultures, normal chest X-rays, and no history of tuberculosis, malignant tumors, and/or HIV.

The study received approval from the Medical Ethics Committee of Anhui Medical University (20,200,250), and informed consent was obtained from each study participant. Peripheral blood samples were collected, along with relevant demographic data, such as age, gender, and clinical signs and symptoms.

Furthermore, it is pertinent to note that this manuscript and our previously published article share the same population. (published article: **“**Association of N6-methyladenosine readers’ genes variation and expression level with pulmonary tuberculosis”).

### SNP selection, DNA extraction, and genotyping

In this study, *THRIL* and *HOTAIR* genes were selected for analysis based on previous research highlighting their potential association with gene polymorphisms and susceptibility to human diseases. Tag SNPs were preferentially selected from the genetic data of CHB in the Ensembl genome browser 85 and CHBS_1000g, with a minor allele frequency (MAF) ≥ 0.05 in CHB. Tag SNPs were chosen through linkage disequilibrium (LD) analysis with an r^2^ threshold > 0.8, employing Haploview 4.0 software (Cambridge, MA, USA). Eventually, two SNPs (rs1055472 and rs11058000) of *THRIL* and four SNPs (rs12427129, rs1899663, rs4759314, and rs7958904) of *HOTAIR* were selected for genotyping.

The genomic DNA was extracted from peripheral blood mononuclear cells (PBMCs) and then isolated using the Flexi Gene-DNA Kit (Qiagen, Valencia, CA). Genotyping was performed using the SNPscan Kit, supported by the Center for Genetic & Genomic Analysis, Genesky Biotechnologies, Inc. (Shanghai). Individuals with a 100% genotyping success rate for the aforementioned SNPs were included in the final analysis.

### Quantitative real-time reverse transcription polymerase chain reaction (qRT-PCR)

PBMCs obtained from 5 ml peripheral blood and stored at − 80 ℃, were processed for RNA extraction using TRIzol Reagent (Invitrogen, Carlsbad, CA, USA). RNA concentration was a NanoDrop 2000 spectrophotometer (Thermo Scientific, USA). The extracted RNA was then reverse-transcribed into cDNA using the PrimeScriptTM RT Reagent Kit (Takara Bio Inc, Japan).

In this study, the expression levels of *THRIL* and *HOTAIR* in PBMCs were assessed through real-time reverse-transcriptase polymerase chain reaction (qRT-PCR) with SYBR Green (SYBR Premix Ex Taq II, Takara Bio Inc, Japan). This experiment was performed using a QuantStudio 12 K Flex real-time PCR system (Applied Biosystems, Foster City, CA, USA). Specific cycling conditions as follows: (1) 1 cycles at 95 °C for 1 min, (2) followed by 42 cycles at 95 °C for 10 s, 60 °C for 30 s, and 72 °C for 1 min. The relative expression levels of lncRNA were calculated using the 2^−ΔΔCt,^ normalized to the endogenous control, with the housekeeping gene β-actin serving as the internal control in the same sample.

### Statistical analysis

All data analyses utilized the Statistical Package of Social Science (SPSS 26.0). The Hardy–Weinberg equilibrium (HWE) of the distribution of all SNP genotypes in healthy controls was assessed using a chi-squared test. Difference in the frequency distribution of SNP genotypes and alleles between different groups were also evaluated with logistic regression analysis, determining Odds ratio (OR) and 95% confidence interval (CI). Two genetic models (dominant and recessive) were analyzed, and haplotype analysis was onducted using SHEsis software. The Mann–Whitney U test was used to analyze the difference in *HOTAIR* and *THRIL* expression levels between the two groups, while Spearman’s rank correlation coefficient test assessed the correlation. A two-sided *P*-value < 0.05 was considered as statistically significant.

## Results

### Relationship between ***THRIL, HOTAIR*** genes polymorphisms and PTB risk

In the genotyping experiment, 456 PTB patients (264 males and 192 females) with an average age of 45.43 ± 17.72 years were compared to a control group of 464 individuals (202 males and 262 females) with an average age of 43.44 ± 13.90 years. The allele and genotype frequencies of the THRIL gene rs1055472 and rs11058000 and HOTAIR gene rs12427129, rs1899663, rs4759314, and rs7958904 are shown in Table 1. In healthy controls, these SNPs exhibited consistency with the Hardy–Weinberg equilibrium test (all *P* > 0.05). The results indicate that no significant differences in allele and genotype distributions of these SNPs polymorphism between PTB patients and controls (all *P* > 0.05). When assessing the association between these SNPs and PTB susceptibility in the dominant and recessive models, no significant differences were found.

**Table 1:** Genotypes and alleles frequencies of THRIL and HOTAIR genes polymorphisms in two groups

SNP	Analyze model		PTB patients	Controls	*P* value	*OR* (95% *CI*)
*THRIL*						
rs1055472	Genotype	AA	80(17.54)	81(17.46)	0.659	0.919(0.630,1.339)
		GA	204(44.74)	223(48.06)	0.271	0.851(0.639,1.134)
		GG	172(37.72)	160(34.48)	Reference	
	Allele	A	364(39.91)	385(41.49)	0.492	0.962(0.862,1.074)
		G	548(60.09)	543(58.51)	Reference	
	Dominant model	GG	172(37.72)	160(34.48)	0.307	1.094(0.921,1.299)
		GA + AA	284(62.28)	304(65.52)	Reference	
	Recessive model	AA	80(17.54)	81(17.46)	0.972	0.999(0.941,1.060)
		GA + GG	376(82.46)	383(82.54)	Reference	
rs11058000	Genotype	AA	13(2.85)	13(2.80)	0.888	1.058(0.482,2.319)
		GA	149(32.68)	140(30.17)	0.407	1.126(0.851,1.490)
		GG	294(64.47)	311(67.03)	Reference	
	Allele	A	175(19.19)	166(17.89)	0.473	1.073(0.886,1.299)
		G	737(80.81)	762(82.11)	Reference	
	Dominant model	GG	294(64.48)	311(67.03)	0.415	0.962(0.876,1.056)
		AA + GA	162(35.53)	153(32.97)	Reference	
	Recessive model	AA	13(2.85)	13(2.80)	0.964	1.018(0.477,2.171)
		GG + GA	443(97.15)	451(97.20)	Reference	
*HOTAIR*						
rs12427129	Genotype	TT	7(1.54)	5(1.08)	0.535	1.442(0.454,4.585)
		CT	84(18.42)	83(17.89)	0.808	1.043(0.745,1.459)
		CC	365(80.04)	376(81.03)	Reference	
	Allele	T	98(10.75)	93(10.02)	0.611	1.072(0.820,1.403)
		C	814(89.25)	835(89.98)	Reference	
	Dominant model	CC	365(80.04)	376(81.03)	0.704	1.052(0.809,1.369)
		CT + TT	91(19.96)	88(18.97)	Reference	
	Recessive model	TT	7(1.54)	5(1.08)	0.541	1.425(0.455,4.456)
		CT + CC	449(98.46)	459(98.92)	Reference	
rs1899663	Genotype	AA	14(3.07)	12(2.59)	0.701	1.167(0.531,2.565)
		CA	149(32.68)	159(34.27)	0.645	0.937(0.711,1.235)
		CC	293(64.25)	293(63.15)	Reference	
	Allele	A	177(19.41)	183(19.72)	0.866	1.004(0.960,1.050)
		C	735(80.59)	745(80.28)	Reference	
	Dominant model	CC	293(64.25)	293(63.15)	0.727	0.970(0.817,1.151)
		GA + CC	163(35.75)	171(36.85)	Reference	
	Recessive model	AA	14(3.07)	12(2.59)	0.658	1.187(0.555,2.539)
		CA + CC	442(96.93)	45(97.41)	Reference	
rs4759314	Genotype	GG	3(0.66)	2(0.43)	0.680	1.459(0.243,8.781)
		GA	47(10.31)	67(14.44)	0.060	0.682(0.458,1.016)
		AA	406(89.04)	395(85.13)	Reference	
	Allele	G	53(5.81)	71(7.65)	0.116	0.760(0.539,1.071)
		A	859(94.19)	857(92.35)	Reference	
	Dominant model	AA	406(89.04)	395(85.13)	0.078	0.737(0.525,1.036)
		GA + GG	50(10.96)	69(14.87)	Reference	
	Recessive model	GG	3(0.64)	2(0.43)	0.984	1.526(0.256,9.092)
		GA + AA	453(99.34)	462(99.57)	Reference	
rs7958904	Genotype	CC	26(5.70)	31(6.68)	0.441	0.805(0.464,1.396)
		CG	179(39.25)	192(41.38)	0.421	0.895(0.684,1.172)
		GG	251(55.04)	241(51.94)	Reference	
	Allele	C	231(25.33)	254(27.37)	0.320	0.925(0.794,1.078)
		G	681(74.67)	674(72.63)	Reference	
	Dominant model	GG	251(55.04)	241(51.94)	0.345	1.060(0.939,1.196)
		CC + CG	205(44.96)	223(48.06)	Reference	
	Recessive model	CC	26(5.70)	31(6.68)	0.538	0.853(0.515,1.414)
		CG + GG	430(94.30)	433(93.32)	Reference	

The findings concerning the associations between all SNPs and major clinical features of PTB patients are shown in Table 2. Notably, the *HOTAIR* rs7958904 CC genotype and C allele exhibited a significant increase in PTB patients with leukopenia compared to those without leukopenia. Conversely, other SNPs of the *HOTAIR* gene and *THRIL* genes showed no significant associations with the clinical features of PTB patients.

**Table 2:** The associations between LncRNA HOTAIR and THRIL genes polymorphisms and clinical features of PTB patients

SNP	Allele	Clinical features	Group	Genotypes n (%)			*P* value	Alleles n (%)		*P* value
	(M/m)			MM	Mm	mm		M	m	
*THRIL*										
rs1055472	G/A	fever	+	24(34.29)	39(55.71)	7(10.00)	0.075	87(62.14)	53(37.86)	0.589
			-	148(38.34)	165(42.75)	73(18.91)		461(59.72)	311(40.28)	
		drug resistance	+	30(36.14)	30(36.14)	23(27.71)	0.770	90(54.22)	76(45.78)	0.186
			-	142(37.08)	174(45.43)	67(17.49)		458(59.79)	308(40.21)	
		DILI	+	31(46.27)	26(38.81)	10(14.93)	0.294	88(65.67)	46(34.33)	0.153
			-	141(36.25)	178(45.76)	70(17.99)		460(59.13)	318(40.87)	
		pulmonary infection	+	31(38.27)	39(48.15)	11(13.58)	0.564	101(62.35)	61(37.65)	0.518
			-	141(37.60)	165(44.00)	69(18.40)		447(59.60)	303(40.40)	
		hypoproteinemia	+	14(35.00)	22(55.00)	4(10.00)	0.281	50(62.50)	30(37.50)	0.645
			-	158(37.98)	182(43.75)	76(18.27)		498(59.86)	334(40.14)	
		leukopenia	+	12(40.00)	13(43.33)	5(16.67)	0.964	37(61.67)	23(38.33)	0.796
			-	160(37.56)	191(44.84)	75(17.61)		511(59.98)	341(40.02)	
		sputum smear	+	42(33.60)	62(49.60)	21(16.80)	0.516	146(58.40)	104(41.60)	0.714
			-	111(38.01)	127(43.49)	54(18.49)		349(59.76)	235(40.24)	
rs1105800	G/A	fever	+	38(54.29)	31(44.29)	1(1.43)	0.070	107(76.43)	33(23.57)	0.152
			-	256(66.32)	118(30.57)	12(3.11)		630(81.61)	142(18.39)	
		drug resistance	+	48(65.75)	24(32.88)	1(1.37)	0.708	120(82.19)	26(17.81)	0.644
			-	246(64.23)	125(32.64)	12(3.13)		617(80.55)	149(19.45)	
		DILI	+	42(62.69)	23(34.33)	2(2.99)	0.947	107(79.85)	27(20.15)	0.760
			-	252(64.78)	126(32.39)	11(2.83)		630(80.98)	148(19.02)	
		pulmonary infection	+	50(61.73)	29(35.80)	2(2.47)	0.794	129(79.63)	33(20.37)	0.674
			-	244(65.07)	120(32.00)	11(2.93)		608(81.07)	142(18.93)	
		hypoproteinemia	+	26(65.00)	13(32.50)	1(2.50)	0.989	65(81.25)	15(18.75)	0.917
			-	268(64.42)	136(32.69)	12(2.88)		672(80.77)	160(19.23)	
		leukopenia	+	17(56.67)	13(43.33)	0(0.00)	0.311	47(78.33)	13(21.67)	0.614
			-	277(65.02)	136(31.92	13(3.05)		690(80.99)	162(19.01)	
		sputum smear	+	81(64.80)	41(32.80)	3(2.40)	0.852	203(81.20)	47(18.80)	0.854
			-	189(64.73)	93(31.85)	10(3.42)		471(80.65)	113(19.35)	
*HOTAIR*										
rs12427129	C/T	fever	+	57(81.43)	11(15.71)	2(2.86)	0.525	125(89.29)	15(10.71)	0.990
			-	308(79.79)	73(18.91)	5(1.30)		689(29.25)	83(10.75)	
		drug resistance	+	57(78.08)	16(21.92)	0(0.00)	0.377	130(89.04)	16(10.96)	0.928
			-	308(80.42)	68(17.75	7(1.83)		684(89.30)	82(10.70)	
		DILI	+	55(82.09)	11(16.42)	1(1.49)	0.899	121(90.30)	13(9.70)	0.673
			-	310(79.69)	73(18.77)	6(1.54)		693(89.07)	85(10.93)	
		pulmonary infection	+	67(82.72)	13(16.05)	1(1.23)	0.800	147(90.74)	15(9.26	0.501
			-	298(79.47)	71(18.93)	6(1.60)		667(88.93)	83(11.07	
		hypoproteinemia	+	32(80.00)	7(17.50)	1(2.50)	0.867	71(88.75)	9(11.25)	0.879
			-	333(80.05)	77(18.51)	6(1.44)		743(89.30)	89(10.70)	
		leukopenia	+	20(66.67)	9(30.00)	1(3.33)	0.155	49(81.67)	11(18.33)	0.050
			-	345(80.99)	75(17.61)	6(1.41)		765(89.79)	87(10.21)	
		sputum smear	+	97(77.60)	25(20.00)	3(2.40)	0.716	219(87.60)	31(12.40)	0.500
			-	233(79.79)	55(18.84)	4(1.37)		521(89.21)	63(10.79)	
rs1899663	C/A	fever	+	44(62.86)	23(32.86)	3(4.29)	0.809	111(79.29)	29(20.71)	0.671
			-	249(64.51)	126(32.4)	11(2.850		624(80.83)	148(19.17)	
		drug resistance	+	45(61.64)	27(36.99)	1(1.370	0.495	117(80.14)	29(19.86)	0.879
			-	248(64.75)	122(31.85)	13(3.39)		618(80.68)	148(19.32)	
		DILI	+	46(68.66)	20(29.85)	1(1.49)	0.587	112(83.58)	22(16.42)	0.343
			-	247(63.50)	129(33.16)	13(3.34)		623(80.08)	155(19.92)	
		pulmonary infection	+	54(66.67)	24(29.63)	3(3.70)	0.780	132(81.48)	30(18.52)	0.752
			-	239(63.73)	125(33.33)	11(2.93)		603(80.40)	147(19.60)	
		hypoproteinemia	+	27(67.50)	12(30.00)	1(2.50)	0.898	66(82.50)	14(17.50)	0.651
			-	266(63.94)	137(32.93)	13(3.13)		669(80.41)	163(19.59)	
		leukopenia	+	14(46.67)	15(50.00)	1(3.33)	0.105	43(71.67)	17(28.33)	0.071
			-	279(65.49)	134(31.46)	13(3.05)		692(81.22)	160(18.78)	
		sputum smear	+	75(60.00)	44(35.20)	6(4.80)	0.382	194(77.60)	56(22.40)	0.193
			-	192(65.67)	92(31.51)	8(2.74)		476(81.51)	108(18.49)	
rs4759314	A/G	fever	+	62(88.57)	7(10.00)	1(1.43)	0.685	131(93.57)	9(6.43)	0.734
			-	344(89.12)	40(10.36)	2(0.52)		728(94.30)	44(5.70)	
		drug resistance	+	68(93.15)	5(6.85)	0(0.00)	0.418	141(96.58)	5(3.42)	0.179
			-	338(88.25)	42(10.97)	3(0.78)		718(93.73)	48(6.27)	
		DILI	+	62(92.54)	5(7.46)	0(0.00)	0.538	129(96.27)	5(3.73)	0.265
			-	344(88.43)	42(10.80)	3(0.77)		730(93.83)	48(6.17)	
		pulmonary infection	+	72(88.89)	8(9.88)	1(1.23)	0.773	152(93.83)	10(6.17)	0.828
			-	334(89.07)	39(10.40)	2(0.53)		707(94.27)	43(5.73)	
		hypoproteinemia	+	38(95.00)	2(5.00)	0(0.00)	0.436	78(97.50)	2(2.50)	0.185
			-	368(88.46)	45(10.82)	3(0.72)		781(93.87)	51(6.13)	
		leukopenia	+	27(90.00)	2(6.67)	1(3.33)	0.142	56(93.33)	4(6.67)	0.770
			-	379(88.97)	45(10.56)	2(0.47)		803(94.25)	49(5.75)	
		sputum smear	+	111(88.80)	12(9.60)	2(1.60)	0.304	234(93.60)	16(6.40)	0.972
			-	256(87.67)	35(11.99)	1(0.34)		547(93.66)	37(6.34)	
rs7958904	G/C	fever	+	38(57.58)	26(39.39)	2(3.03)	0.523	102(77.27)	30(22.73)	0.576
			-	213(55.18)	153(39.64)	20(5.18)		579(75.00)	193(25.00)	
		drug resistance	+	41(56.16)	30(41.10)	2(2.74)	0.489	112(76.71)	34(23.29)	0.536
			-	210(54.83)	149(38.90)	24(6.27)		569(74.28)	197(25.72)	
		DILI	+	41(61.19)	24(35.82)	2(2.99)	0.408	106(79.10)	28(20.90)	0.201
			-	210(53.98)	155(39.85)	24(6.17)		575(73.91)	203(26.09)	
		pulmonary infection	+	46(56.79)	29(35.80)	6(7.41)	0.652	121(74.69)	41(25.31)	0.995
			-	205(54.67)	150(40.00)	20(5.33)		560(74.67)	190(25.33)	
		hypoproteinemia	+	26(65.00)	11(27.50)	3(7.50)	0.275	63(78.75)	17(21.25)	0.380
			-	225(54.09)	168(40.38)	23(5.53)		618(74.28)	214(25.72)	
		leukopenia	+	13(43.33)	12(40.00)	5(16.67)	**0.023**	38(63.33)	22(36.67)	**0.037**
			-	238(55.87)	167(39.20)	21(4.93)		643(75.47)	209(24.53)	
		sputum smear	+	63(50.40)	51(40.80)	11(8.80)	0.309	177(70.80)	73(29.20)	0.188
			-	162(55.48)	115(39.38)	15(5.14)		439(75.17)	145(24.83)	

### Haplotype analysis

Haplotype analysis was conducted using the SHEsis software, examining *THRIL and HOTAIR* genes. Data were excluded when the frequency was < 0.03 in both PTB patients and healthy controls. Three primary haplotypes (AA, AG, and GG) for THRIL and four main haplotypes (CAAC, CCAG, CCGC, and TAAC) were identified in our study (Table 3). However, no significant differences were observed in the frequencies of these haplotypes between PTB patients and healthy controls.

**Table 3:** Haplotype Analysis of LncRNA THRIL and HOTAIR Genes in PTB Patients and Controls

Haplotype	PTB [n(%)]	Controls [n(%)]	*P* value	*OR* (95% CI)
*THRIL* rs1055472-rs11058000				
AA	174.99(19.2)	164.69(17.7)	0.434	1.099 (0.868,1.391)
AG	189.01 (20.7)	220.31(23.7)	0.116	0.838(0.673,1.045)
GG	547.99(60.1)	541.69(58.4)	0.476	1.070(0.888,1.289)
*HOTAIR* rs12427129- rs1899663- rs4759314-rs7958904				
CAAC	79.12(8.7)	90.00(0.097)	0.417	0.877(0.639,1.204)
CCAG	681.00(74.7)	670.55(72.3)	0.364	1.101(0.894,1.357)
CCGC	53.00(5.8)	68.10(7.3)	0.173	0.773(0.533,1.120)
TAAC	97.88(10.7)	91.31(9.8)	0.565	1.093(0.808,1.476)

*Frequency < 0.03 in both controls & PTB patients has been dropped

### ***THRIL and HOTAIR*** gene expression levels in PTB patients and normal controls

**Fig. 1:**
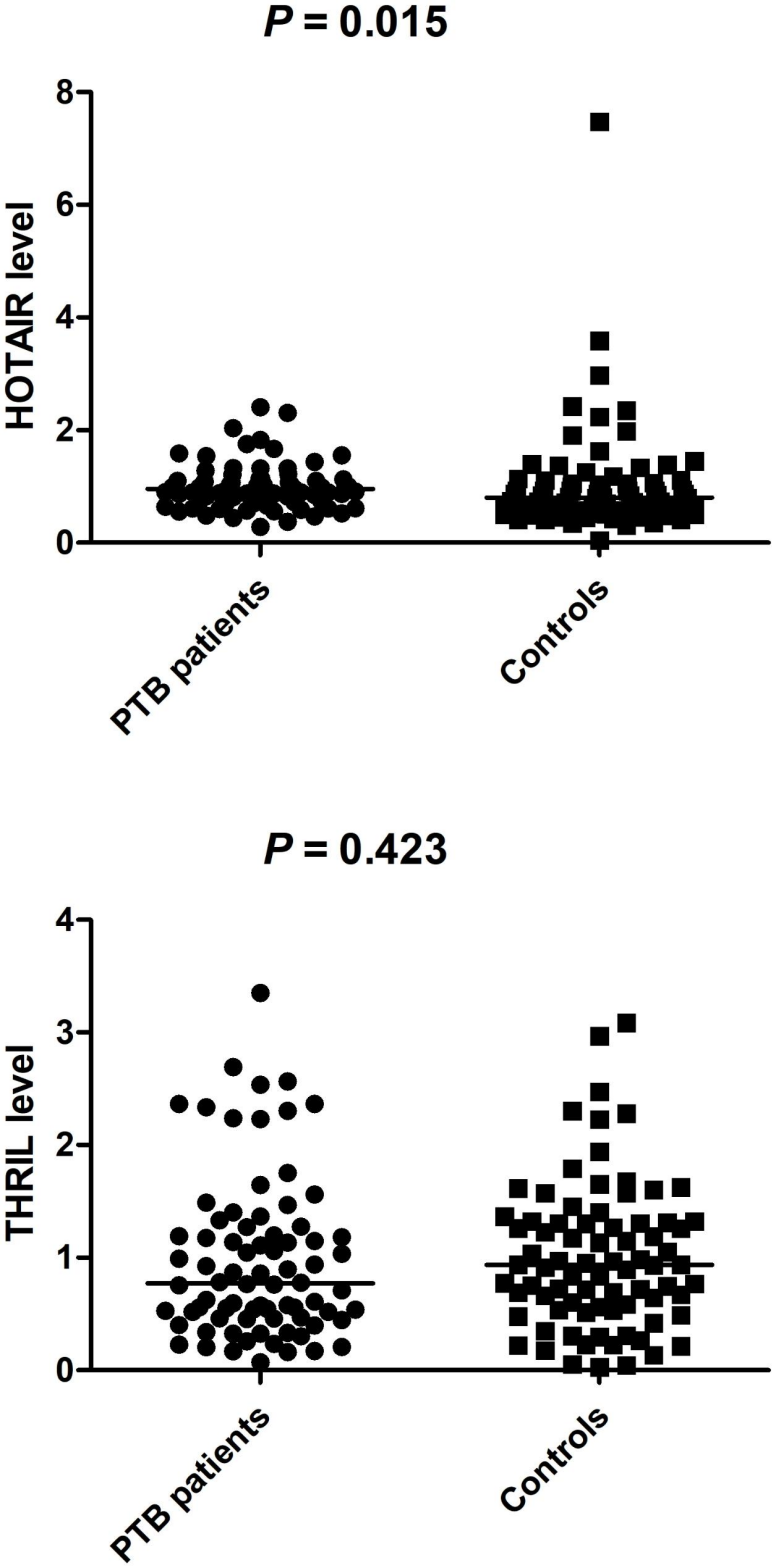
LncRNA THRIL and HOTAIR gene expression levels in PTB patients and normal controls

A comprehensive analysis was performed to assess the correlation between mRNA expression levels in the PBMCs of PTB patients and normal controls. Figure 1 illustrates that the expression levels of the *HOTAIR* gene in patients were significantly higher than those in normal controls (*P* = 0.015). However, no significant difference in THRIL gene expression levels between these two groups was identified (*P* > 0.05).

We also conducted an alysis to investigate the association between the expression levels of these two lncRNA’s and certainclinical symptoms of PTB patients. Subsequently, we observed that the mRNA level of HOTAIR and THRIL genes exhibited no discernible correlation with the occurrence of clinical features, including fever, drug resistance, liver injury, lung infection, hypoproteinemia, leukopenia, and positive sputum smear in PTB patients (Table 4). Similarly, the expression levels of these two genes demonstrated no significant correlation with the erythrocyte sedimentation rate, total bilirubin, aspartate transaminase, alanine transaminase (ESR, TBIL, AST, and ALT, respectively) in PTB patients (Table 5).

**Table 4:** The Association Between lncRNA *THRIL* and HOTAIR Expression Levels and Several Clinical Features in PTB Patients

Group	+/-*	N	*HOTAIR* level	*P* value	*THRIL* level	*P* value
Fever	+	15	0.943(0.802,1.104)	0.785	0.780(0.559,1.200)	0.392
	-	63	0.966(0.705,1.133)		0.761(0.455,1.275)	
Drug-resistant patients	+	5	0.912(0.774,1.717)	0.797	0.596(0.280,1.330)	0.634
	-	73	0.966(0.730,1.120)		0.779(0.464,1.274)	
Liver injury	+	8	0.882(0.836,0.914)	0.162	0.542(0.449,1.184)	0.449
	-	70	0.991(0.723,1.136)		0.780(0.465,1.340)	
pulmonary infection	+	11	1.002(0.879,1.224)	0.523	1.137(0.460,1.753)	0.651
	-	67	0.920(0.724,1.107)		0.764(0.457,1.200)	
hypoproteinemia	+	16	0.896(0.678,1.072)	0.665	0.717(0.449,1.967)	0.902
	-	62	0.976(0.733,1.134)		0.772(0.465,1.273)	
leukopenia	+	6	1.115(0.754,1.771)	0.368	0.577(0.417,0.785)	0.230
	-	72	0.955(0.727,1.020)		0.825(0.462,1.132)	
Sputum smear-positive	+	27	1.002(0.736,1.140)	0.546	0.75590.460,1.183)	0.937
	-	51	0.943(0.705,1.087)		0.779(0.401,1.275)	

*+/-: with/without; median (interquartile range);

**Table 5:** The correlation between lncRNA *THRIL* and *HOTAIR* expression levels and ESR, TBIL, ALT, AST of PTB patients

Clinical parameters	*HOTAIR* level		*THRIL* level	
	rs*	*P* value	*rs*	*P* value
ESR	-0.157	0.177	-0.085	0.467
TBIL	-0.088	0.451	0.029	0.805
ALT	-0.005	0.968	-0.05	0.663
AST	-0.156	0.177	-0.053	0.650

*rs: Spearman’s rank correlation coefficient

### LncRNA genes polymorphisms and their expression levels in PTB patients

We conducted analysis of genotype frequencies and expression levels of *HOTAIR* and *THRIL* genes in 78 patients with PTB. However, the expression levels of these genes did not exhibit significant difference between PTB patients with different genotypes (Table 6).

**Table 6:** Association Between LncRNA *THRIL* and *HOTAIR* Genes Polymorphisms with Their Expression levels in PTB Patients

*THRIL* SNP	Genotype	number	*THRIL* level*	*P* value
rs1055472	AA	14	0.760(0.382,0.987)	0.353
	GA	25	1.044(0.505,1.319)	
	GG	23	0.557(0.400,1.149)	
Rs11058000	AA	3	0.460(0.401,3.350)	0.999
	GA	19	0.780(0.416,1.252)	
	GG	40	0.695(0.210,0.700)	
*HOTAIR* SNP	Genotype	number	*HOTAIR* level	*P* value
rs12427129	CC	49	0.966(0.805,1.082)	0.952
	CT	13	0.993(0.505,1.498)	
	TT	0	0	
rs1899663	CC	36	0.955(0.812,1.100)	0.651
	CA	26	0.991(0.597,1.182)	
	AA	0	0	
rs4759314	AA	56	0.976(0.709,1.127)	0.407
	GA	6	1.010(0.881,1.337)	
	GG	0	0	
rs7958904	GG	31	0.917(0.802,1.087)	0.830
	GC	28	1.010(0.617,1.182)	
	CC	29	0.885(0.885,0.885)	

*Median (interquartile range)

## Discussion

In recent years the identification of numerous lncRNAs with a variety of biological functions have been identified in humans has expanded significantly [12]. Extensive research has highlighted the vital roles played by lncRNAs in the progression of various diseases, such as tumors, autoimmune diseases, cardiovascular disorders, neurodegenerative diseases, and others [22–25]. A study by Dang et al. demonstrated that lncRNA-ATB (long non-coding RNA activated by transforming growth factor-β) is implicated in osteoarthritis pathogenesis via activation of adenylate kinase (AK) signaling and regulation of chondrocyte proliferation and activity [26]. Notably, down-regulation of lncRNA-ATB in serum appears to be a credible diagnostic indicator of osteoarthritis [26]. Similarly, the regulatory roles of lncRNA are also crucial in PTB. Sun et al. demonstrated that lncRNA NORAD elevated significantly in PTB patients, influencing macrophage activity and inflammation of MTB infection by targeting Mir-618 [27]. These findings underscore the potential of lncRNA polymorphisms as novel molecular biomarkers for diagnosis of various human diseases, including PTB. However, only limited research exists on LncRNA gene polymorphisms and PTB risk, prompting this study to investigate the association between lncRNA THRIL and HOTAIR genes variations and expression levels with PTB.

Several studies proposed that *HOTAIR* and *THRIL* could serve as potential new molecular biomarkers for tumors through regulation of various genes [28, 29]. For instance, Zhang et al. found that the expression of *HOTAIR* in the plasma of breast cancer patients was surpassed that of healthy controls, exhibiting higher diagnostic ability and specificity than that of CA153 and CEA [30]. Another study revealed significantly elevated expression levels of HOTAIR in colon cancer tissue compared to matched normal colon tissue adjacent to the cancer, indicating its potential as a molecular target for colon cancer treatment [31]. Furthermore,,*HOTAIR* and *THRIL* play crucial roles in regulating immune activity and inflammation responses [19, 23]. Subuddhi et al. reported early downregulation of *HOTAIR* in MTB H37Rv infection, marking the first observationof *HOTAIR’s* role in regulating immune responses in MTB [32]. Similarly, it was observed that maternal *HOTAIR* rs4759314AG genotype and *HOTAIR* rs4759314 polymorphism in dominant and allelic models were associated with increased risk of PE [21]. However, the involvement of *THRIL* and *HOTAIR* genetic polymorphisms are involved in the pathophysiological processes of PTB remains unexplored. Addressing this,, we initiated an analysis of the association between the THRIL gene (rs1055472 and rs11058000) and HOTAIR gene (rs12427129, rs1899663, rs4759314, and rs7958904) polymorphisms and risk of PTB. Unfortunately, we did not observe any significant association between these SNPs in both THRIL and HOTAIR and PTB susceptibility. Patients with PTB often present multiple and complex clinical manifestations, including fever, lung infection, hypoproteinemia, hypoproteinemia among other signs and symptoms, which could be influenced by genetic variations [3, 16]. Wu et al. identified a significant association between Lnc-HNF1B-3:1 rs12939622, rs4262994 and fever in PTB patients [14]. Similarly, in our previous study, we observed a significant correlation between the NEAT1 gene rs3825071 variant TT genotype and T-allele frequencies were significantly related to positive sputum smears in PTB patients [16]. In this study, we obtained a comparable result indicating a significant relationship between C allele frequencies in the *HOTAIR* gene rs7958904 variant CC genotype, C allele frequencies and leukopenia of PTB patients. These findings suggest that gene variants may play a role in PTB development, whereby contributing valuable insights for the formulation of effective treatment strategies.

Increasing evidence highlights the involvement of expression level of multiple lncRNA in the pathogenesis and progression of PTB [33]. Ye et al. reported elevated plasma lncRNA CCTAT1 levels in newly developed TB patients compared to patients with recurrent TB and healthy controls, and high CCAT1 levels correlated with increased mortality in TB patients [34]. Another study demonstrated a specific and significant upregulation expression levels of lncRNA AC700128.1 in TB patients [15], emphasizing existence of differential expression of lncRNA in PTB. Consequently,, we analyzed the expression levels of HOTAIR and THRIL in PBMCs of case group and healthy controls. Our findings revealed significantly higher levels of *HOTAR* in PTB patients than in healthy controls, while *THRIL* levels did not exhibit any statistical significance. This finding implies the potential involvement of *HOTAR* in the development of PTB and proposes elevated expression levels of *HOTAR* as a promising diagnostic biomarker for PTB. Subsequently, We further explored the association between expression levels of HOTAIR and THRIL and clinical features in PTB patients, however no significant results were detected.

In conclusion, the present study found that *HOTAIR* rs7958904 gene polymorphism can be serve as a suggested biomarker to find patients with Leukopenia. However, no association was observed between *THRIL* rs1055472 and rs11058000 and *HOTAIR* rs12427129, rs1899663, rs4759314 and rs7958904 polymorphisms and PTB risk. In addition, compared with the healthy controls, levels of HOTAIR in case group were significantly higher, suggesting its potential use as an auxiliary biomarkers for PTB diagnosis. Nonetheless, our study has some limitations. Firstly, the sample size may be insufficient, which might lead to inaccurate data results. Secondly, factors such as ethnic background, disease course, treatment, and other may have influenced the study outcomes. Therefore, further research with a larger sample size and diverse ethnic backgrounds is warranted to better define the potential roles of *HOAIR* and *THRIL* in PTB.

## Data Availability

The data generated and analyzed for this study are available from the corresponding author upon reasonable request.
